# Carbolized Jute as a Wound Dressing

**Published:** 1879-05-20

**Authors:** Robert F. Weir

**Affiliations:** Surgeon to the Roosevelt and New York Hospitals


### *ON CARBOLIZED JUTE AS A WOUND-DRESSING.*

BY ROBERT F. WEIR, M.D.,

Surgeon to the Roosevelt and New York Hospitals.

In the use of Lister's carbolized cotton gauze several objections are found, which materially interfere with the more general use of this dressing in the treatment of wounds. The disadvantages are, first, the difficulty of its manufacture. This of itself often prevents a surgeon, uuless living within reach of a large city where the gauze is most likely to be made,, from undertaking a trial of the dressing, or is a reason for his not continuing it even when once resorted to.

Without having some impregnated gauze on hand, it is evident that recourse to the antiseptic treatment is not readily to be had; for, the use of lint, muslin, cotton, or other substances, impregnated with a watery solution of carbolic acid, is, by reason of the rapid evaporation of the acid (95 per cent. at the temperature of the body in the course of 24 hours), very unreliable; indeed, when such contingencies arise, carbolized olive oil (1–20) makes a much more serviceable dressing, and should be employed. Another reason for discouragement in the use of the gauze comes from the frequent inability, for anatomical reasons, to secure this dressing properly *in situ*. Such difficulty exists, for example, about the male genitals, where it is necessary to leave an opening in the dressing for the penis to emerge; also about the groin; also on the upper part of the chest walls, or in the axilla, as after mammary ablations, etc. Although any gaping of the dressing is remedied to a certain extent by the use of rubber bandages lightly applied, by fastening the edges of the dressing with collodion, or by stuffing in salicylized cotton, yet those having experience in the Lister method will admit that more care and anxiety are demanded in connection with such cases than in injuries elsewhere located. The third objection is the expense of the carbolic gauze.

Now these objections are well met by the use of the carbolized jute. Jute, as is well known, is the inner bark of a Bengal plant, the *corchoris capsularis*, and is an extremely absorbent substance. It has been salicylized and used with a certain success by Thiersch; it has also been used oy Bardeleben in the form of large masses or "cakes," dipped in an aqueous solution of carbolic acid. But it is only recently that a way of impregnating it, or the like substance, tow, with carbolic acid, has been found, and for this improvement we are indebted to Munnich, a German surgeon, who, in the Deutsche Militairarztliche Zeitschrift, No. 10, 1877, published an article, not only setting forth how this could be done, but also sundry advantages accruing from the use of prepared jute in military surgery.

The trouble that has hitherto been experienced is to properly proportion the amount of resin necessary to cause the slow evolvement of the carbolic acid, and yet to prevent the jute fibres being gummed together into a sticky mass. Prepared, however, by the following formula, Munnich accomplished the successful solution of the question.

He takes for 1℔ (500 grammes) of jute :

| | | |
|---|---|---|
| 50 grammes | (ʒxiij) | of carbolic acid. |
| 200 " | (ʒl) | " resin. |
| 250 " | (ʒlxij) | " glycerine. |
| 550 " | (ʒcxxxviij) | " alcohol. |

These are mixed together in the following manner: The finely pulverized resin is first dissolved in the larger part of the alcohol with the aid of heat gently applied: after cooling this the carbolic acid, which has been dissolved in the remaining portion of the alcohol, is added, and, after some minutes, the glycerine. The solution is then poured on the jute and worked up with it, so as to thoroughly moisten all its fibres, and when the mass begins to adhere together, from evaporation of the alcohol, it is carded or pulled apart, and spread out to dry. The carding, which is to prevent the adhesion of the fibres, becomes very easy if 50 grammes of stearine are dissolved with the resin and added to the mixture. But in this case the jute dries somewhat more slowly. Munnich further states that, without the addition of the stearine, the jute will dry in four hours, and will be ready for use in from twelve to eighteen hours thereafter, and that it will keep best when strongly compressed, wrapped in parchment paper, and placed in a closed box. A good preparation should be entirely homogeneous, have a strong carbolic odor, and the fibres should not be moist, nor adhere on firm pressure.

Jute prepared in this way, without the stearine, I have used in the Roosevelt and New York Hospitals for nearly a year, and, up to the present time, it has been tried in 67 cases of various injuries and operations.

Jute is used now in the above-named hospitals in the place of the carbolized gauze; instead, however, of omitting the protective, as Munnich does, this is laid as usual over the line of incision, and over it the jute is applied in a layer one or two inches thick, but extending to the same distance from the wound as in the gauze dressing. The rubber cloth or mackintosh, used by Lister to cause the secretions to flow towards the edge of the dressing, as well as to hinder evaporation, is placed over the jute and secured by a bandage. Sometimes the jute is placed between two layers of gauze, and underneath the outer one the mackintosh is inserted. This dressing fits with snugness, is very absorbent, differing in this respect from oakum or tow, which collects, as is well known, the discharge of the surface, and not only is readily made, but is very cheap. This latter item is particularly of importance in connection with the employment of antiseptic dressings in hospitals. In reality, the cost of the Lister dressing is of serious moment, and is now occupying the attention of the governing boards of a number of the institutions in which its use is rapidly and satisfactorily increasing.

But through the kindness and skill of Mr. Rosenwasser, apothecary to the New York Hospital, I have been able to make a preparation of carbolized jute of the strength of 7.2 per cent. of acid, after drying, that costs but fifteen cents per pound. This is accomplished mainly by the use of benzine instead of alcohol in its preparation.

The formula is as follows: For 1℔ (or 7000 grains, avoirdupois) of jute, take:

| | |
|---|---|
| Crystalized carbolic acid................... | 700 grains. |
| Paraffin.................................. | 700 " |
| Resin...................................... | 2800 " |
| Benzine.................................... | 3 pints. |

Or, in other words, for a pound of jute—10 per cent. of carbolic acid is required, 40 per cent. of resin, and 10 per cent of paraffin, with enough benzine to thoroughly moisten. The larger the quantity of jute impregnated at one time, the smaller in proportion is the requisite quantity of benzine.—*Amer. Journal of Med. Science.*

